# Novel LMNA mutations in Greek and Myanmar Patients with Progeroid Features and Cardiac Manifestations

**DOI:** 10.31491/apt.2020.06.021

**Published:** 2020

**Authors:** Renuka Kandhaya-Pillai, Fuki M. Hisama, Stephanie A. Bucks, Soe Yarzar, Haroula Korovou, George M. Martin, Junko Oshima

**Affiliations:** a Department of Pathology, University of Washington, Seattle, WA, USA.; b Division of Medical Genetics, Department of Medicine, University of Washington, Seattle, WA, USA.; c Department of Laboratory Medicine, University of Washington, Seattle, WA, USA.; d Department of Medicine, University of Medicine 2, Yangon, Myanmar.; e Phaze SA, Athens, Greece.

**Keywords:** Lamin A/C, atypical Werner syndrome, progeroid syndrome, medical genetics, human

## Abstract

Segmental progeroid syndromes are groups of genetic disorders with multiple features resembling accelerated aging. The International Registry of Werner Syndrome (Seattle, WA) recruits pedigrees of progeroid syndromes from all over the world. We identified two novel LMNA mutations, p.Asp300Gly in a patient from Myanmar, and p.Asn466Lys, in a patient from Greece. Both were referred to our Registry for the genetic diagnosis because of the accelerated aged-appearance and cardiac complications. LMNA mutations are the second most common genetic cause of progeroid syndromes after WRN mutations in our Registry. As the next generation sequencing becomes readily available, we expect to identify more cases of rare genetic diseases in the developing countries.

## Introduction

Laminopathies are a group of genetic disorders caused by mutations at the LMNA locus. The LMNA gene encodes nuclear intermediate filaments, lamin A and C (lamin A/C), generated by alternative splicing [1, 2]. Lamin A/C proteins consist of an N-terminal globular domain, α-helical coil domains, a nuclear localization signal, and a C-terminal globular domain. Lamin A, but not lamin C, has a C-terminal tail end that undergoes successive post-translational modifications for the maturation from prelamim A to lamin A. Lamin A/C, as well lamin B encoded by the LMNB1 gene, are components of the nuclear lamina, a protein network structure underlying the inner nuclear membrane [1, 3, 4]. In addition to the structural maintenance of nuclei, nuclear lamina also plays diverse roles in chromatin organization, intracellular signaling, and transcriptional regulations [2, 4–6].

There are two major groups of disease mutations of the LMNA gene. One consists of missense mutations, which are thought to affect the dimerization of lamin A/C and/or to perturb intermolecular interactions in nuclear lamina [2, 4]. Diseases associated with substitution mutations include isolated disorders or overlapping syndromes of dilated cardiomyopathy, muscular dystrophies, lipodystrophies, mandibuloacral dysplasia, Charcot-Marie Tooth neuropathy and progeroid syndromes [1, 2, 7]. The second group consists of heterozygous substitution at the junction of exon 11 and intron 11, which activate cryptic splice sites and in-frame deletions including the region of proteolytic site required for the maturation of lamin A. The resultant mutant form of lamin A, termed progerin, is responsible for the Hutchinson-Gilford progeria syndrome and, depending on the level of progerin, a milder form of progeria [8, 9]. To date, nearly 500 different mutations have been identified across the LMNA gene (The UMD-LMNA mutations database, www.umd.be/LMNA).

Here, we report new cases of LMNA mutations from Myanmar and Greece. Both suffer from cardiac complications and were referred to the International Registry of Werner Syndrome (www.wernersyndrome.org) because of their striking appearances of accelerated aging.

## Case reports

### Case 1

**Figure 1.**
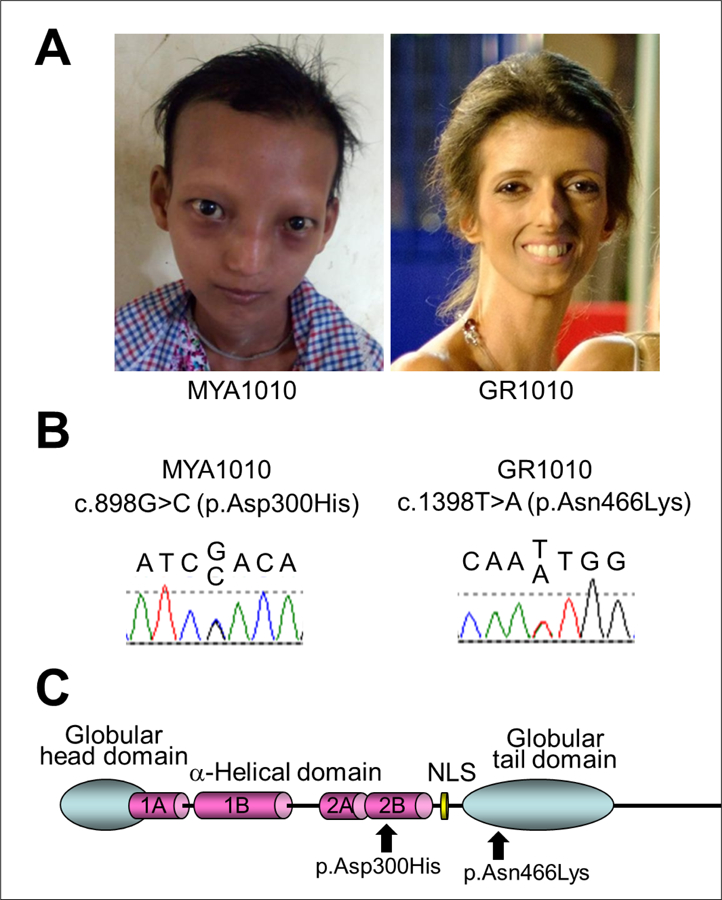
LMNA mutations in progeroid syndrome. A. Facial profiles of MYA1010 (left) and GR1010 (right). B. Sanger confirmation of the LMNA mutations in MYA1010 (left) and GR1010 (right). C. Functional domains of lamin A protein and the locations of the LMNA mutations identified in this study.

Registry#MYA1010 (Figure 1A) is a 23-year old woman from a remote part of Myanmar who was well until age 18 years, when she was diagnosed with hypertension, and began to have graying of her hair and changes of the skin of her face and hands. Her menarche was at 18 years, but she developed secondary amenorrhea. At age 23 years, she was brought to medical attention for a two week history of dyspnea on exertion. On examination, she was very short-statured: height 130 cm (Z score −5), weight was 24 kg (Z score −11). She was hypertensive, with blood pressures of 170/90 from the right arm and 230/100 from the left arm, and exhibited a carotid bruit. She had generalized lipoatrophy with tight skin of the face and hands. She had a high-pitched voice, and examination of the head revealed dysmorphic features: a tall forehead, thinning scalp hair, absent eyebrows and eyelashes, hypertelorism, and a beaked nose. Cardiovascular exam revealed a carotid bruit, systolic murmur at the lower left sternal border and a loud S2. Secondary sex characteristics were poorly developed. Her limbs were extremely thin. An ophthalmological evaluation revealed retinal hemorrhages, and the absence of cataracts (a cardinal sign of the Werner syndrome).

A complete family history is unavailable. She is an only child, and her mother died at 25 years of age after a fever. Her father is absent. Her ethnicity is Kayin, from Myanmar.

Routine laboratory testing revealed normal results for complete blood counts, and blood chemical parameters, including fasting blood glucose, and lipids. A panel of testing for 23 antibodies associated with rheumatologic diseases, including scleroderma, was negative. Chest X ray revealed a left sided pulmonary effusion. Carotid Doppler exam revealed bilateral carotid artery stenosis. An echocardiogram revealed mild concentric left ventricular hypertrophy, with ejection fraction 67%, no regional wall motion abnormalities, a thickened, calcified mitral valve, moderate mitral stenosis, and pulmonary hypertension. A CT aortogram revealed a normal aorta, without evidence of stenosis or dilatation, and normal iliac, mesenteric and renal arteries.

### Case 2

Registry#GR1010 was born to non-consanguineous parents from Greece. Her early development was said to have been normal. At age 26, she was evaluated for a thyroid nodule, which was diagnosed as a well-differentiated papillary thyroid carcinoma, for which she underwent thyroidectomy and ablation with radioactive iodine. At age 35, she was diagnosed with premature ovarian insufficiency. An ophthalmological evaluation was negative for cataracts. Her blood pressure was normal, but she had severe cardiac valve calcifications.

At age 37 years her height was a normal 163 cm (48^th^ %tile, Z score −0.05), weight was 35 kg (<1st %tile, Z score −4.6). She had a progeroid appearance with thin, tight, skin, lipoatrophy, alopecia, and cafe au lait macules. Her neurodevelopmental history was said to have been normal. A notable feature of this examination was skeletal muscle atrophy.

She was hospitalized at age 37 years for myocardial infarction. She was found to have atherosclerosis, and osteoporosis. She died from cardiovascular complications. A progeroid syndrome was suspected by her treating physicians, and the International Werner Syndrome Registry was contacted to establish a diagnosis.

A family history was negative for relatives with progeroid features. The paternal height was 175 cm (40th %tile, Z score −0.26) and the maternal height was 162 cm (42^nd^ %tile, Z score −0.21). She has two apparently normal brothers.

### Identification of LMNA mutations

Exome sequencing of Registry# MYA1010 revealed a heterozygous variant in the exon 5 of the LMNA gene, c.898G>C, p.Asp300His, which was confirmed by the Sanger sequencing (Figure 1 B&C). This variant has a CADD score of 35 with a Polyphen-2 prediction that it was pathogenic; the Polyphen score was 0.96, indicating a highly significant mutational change [10, 11]. In the ClinVar database, p.Asp300Gly is listed as pathogenic and p.Asp300Asn is listed as likely pathogenic, although p.Asp300His is not listed. Parental samples were not available for testing.

Sanger sequencing of LMNA exons in Registry# GR1010 showed a heterozygous variant, c.1398T>A, p.Asn466Lys, in exon 8 (Figure 1 B&C). The CADD score of this variant was 20 and the Polyphen-2 prediction was benign (score 0.42). Previously, the Popyphen-2 program predicted a well-described lipodystrophy mutation, p.Arg482Gln (rs11575937), to be benign (score 0.069) and our previously described lipodystrophy mutation, p.Pro485Arg, also to be benign (score 0.11) [12], emphasizing the limitations of some current prediction programs. LMNA variants at amino acid position 466 were not listed in ClinVar. Parents were not tested for this variant.

In both patients, we did not find pathogenic variants characteristic of two other known progeroid loci, WRN or POLD1. Taken together, we concluded that we had identified novel heterozygous LMNA variants as likely genetic causes of the progeroid syndromes in both patients described above.

## Discussion

We identified two independent cases of laminopathy with novel heterozygous LMNA mutations, p.Asp300His and p.Asn466Lys. Pathogenic variants at amino acid position 300 have previously been reported in multiple patients, including p.Asp300Asn in a case of “atypical Werner syndrome” with acute ischemic cerebral disease [13] and p.Asp300Gly in a case of “atypical progeroid syndrome” [7]. Different pathogenic variants at position 466, p.Asn466Asp, have also been reported in the Dunnigan type of familial partial lipodystrophy [14]. We concluded that these identified variants are responsible for the conditions of these two patients. Based on the guideline of The American College of Medical Genetics and Genomics (ACMG), both variants found in our patients are classified as Likely Pathogenic based on the combined criteria (category: PM2+PM5+PM6+PP3) [15]. The underlying molecular mechanisms, however, remain to be established. There is a possibility of the second modifying loci not having been picked up by current exome analysis.

The International Registry of Werner Syndrome (www.wernersyndrome.org) recruits a wide range of segmental progeroid syndromes from around the world with the goal of elucidating underlying mechanisms of accelerated aging. In our Registry, LMNA accounts for 7% (15/223) of the genetically diagnosed cases [9, 12, 16, 17], and is the second most common progeroid locus after WRN, which accounts for 82% (183/223) [18]. That figure, however, likely reflects a bias of ascertainment, as our Registry was initially developed in order to map and clone the responsible locus underlying the Werner syndrome.

LMNA mutations are thought to be among the most common causes of familial dilated cardiomyopathy, accounting for approximately 10% [2, 19–21]. Our cases are from the regions of Myanmar and Greece, where genetic testing is not routinely performed. It is likely that laminopathies as well as other progeroid syndromes are underdiagnosed in these areas. As next generation sequencing becomes readily available, we expect to find more cases of these rare genetic disorders.
